# USF Age Dissemination Project: Bridging the Gap for Healthy Aging Through Academic Research

**DOI:** 10.1093/geroni/igaf122.2509

**Published:** 2025-12-31

**Authors:** Jessica VanderWerf, Natalia Babenko, Lindsay Peterson, Kathy Black

**Affiliations:** University of South Florida, Tampa, Florida, United States; University of South Florida, Tampa, Florida, United States; University of South Florida, Tampa, Florida, United States; University of South Florida, Tampa, Florida, United States

## Abstract

Academic research can be difficult for older adults to access, creating barriers to raising awareness about healthy aging and improving quality of life in later years. Limited access means that older individuals may miss out on insights into disease prevention, mental well-being, and lifestyle interventions that could enhance their longevity and independence. Disseminating research in an accessible and engaging manner is essential, as it empowers older adults with knowledge, informs caregivers and policymakers, and promotes the adoption of evidence-based practices that support aging with dignity and well-being. The USF Age Dissemination Project seeks to bridge this gap by enhancing academic outreach while providing a replicable model for other universities. This initiative employs sustainable methods for distributing research to communities of older adults in a format that is both understandable and practical. This poster presents the early stages of this project, outlining its framework and offering guidelines for other institutions looking to implement similar initiatives. Specifically, the project involves the development of a product called “News We Can Use to Age Well”, a structured template designed to disseminate research to older adults outside academia. The poster describes the initial steps of the process, which involved evaluating articles, analyzing them for accessibility, and translating them into an age-friendly format that is easy to understand and apply. The poster also discusses future steps aimed at expanding the project’s reach, impact, and community engagement with university research. By improving access to knowledge, this initiative aims to empower older adults and support healthy, informed aging.

